# Hepatic Lesions with Secondary Syphilis in an HIV-Infected Patient

**DOI:** 10.1155/2014/604794

**Published:** 2014-10-02

**Authors:** Paola R. Solari, Christopher Jones, Mark R. Wallace

**Affiliations:** Orlando Health, 1414 Kuhl Avenue, Orlando, FL 32806, USA

## Abstract

Syphilis among HIV-infected patients continues to be a public health concern, especially in men who have sex with men. The clinical manifestations of syphilis are protean; syphilitic hepatitis is an unusual complication that can occur at any stage of the disease. We report a case of an HIV-infected male who presented with systemic symptoms and liver lesions highly suggestive of lymphoma and was proven to have syphilitic hepatitis by liver biopsy. Our case reinforces the importance of recognizing syphilis as a possible cause of unexplained abnormal liver enzymes and/or hepatic lesions in HIV-infected patients.

## 1. Introduction

Syphilis among HIV-infected patients continues to be a public health concern. Although liver involvement with early syphilis is an infrequently recognized complication, it is an especially challenging diagnosis in HIV-infected individuals who often have abnormal liver function tests from any one of multiple potential causes [1]. Coexisting conditions such us hepatitis C, hepatitis B, mycobacterial infection, and neoplastic diseases as lymphoma or Kaposi's sarcoma may cause liver enzymes abnormalities in this population, as well as medications, substance abuse, and fatty liver disease.* Treponema pallidum* is known to cause hepatitis, but relatively few case reports have been published of hepatic involvement in early syphilis in HIV-infected patients [1–4]. We present a case of an HIV positive male with hepatic lesions suggestive of lymphoma who was eventually diagnosed with syphilitic hepatitis after liver biopsy.

## 2. Case Presentation

A 59-year-old married male with well-controlled insulin dependent diabetes mellitus, tinea versicolor, and long term nonprogressive HIV infection presented to our clinic with five weeks of generalized muscle, joint and back pain, headache, and weight loss of about seven pounds over three weeks. Two months prior to this presentation, at his annual HIV restaging, his CD4 was 650 cells/*μ*L and quantitative HIV load was 481 copies/mL. His CD4 counts and viral loads had been in this range for eighteen years without any antiretroviral therapy. At the same routine annual visit, his urine screening for chlamydia and gonorrhea was negative, a RPR was nonreactive, and a hepatitis panel showed immunity to hepatitis B without evidence of acute or chronic viral hepatitis. A chest radiograph was normal. His hemoglobin A1c was 7.7%.

On examination he was afebrile and had no palpable lymph nodes, hepatosplenomegaly, or neurologic deficits. He did have a hypopigmented, macular rash limited to his back consistent with tinea versicolor. Serologies for cytomegalovirus, parvovirus B-19, and Epstein-Barr virus were consistent with past infection. No specific therapy was given. He returned to clinic two months later with persistent symptoms of headache, myalgias, and back pain. He now reported, additionally, that he had developed fever, sweats, blurred vision in his left eye, alopecia, a moccasin-type erythrodermic rash on his feet, and additional 10 pound weight loss. He denied any sexual activity for greater than 5 years, including oral sex. He had seen an optometrist for his blurry vision and been diagnosed with a left cataract; another physician he visited had prescribed an antifungal for his foot rash without improvement. Abdominal examination revealed new tenderness in the right upper quadrant with hepatomegaly. Laboratory investigation showed CD4 of 369 cells/*μ*L and HIV load of 1314 copies/mL. His alkaline phosphatase was elevated at 209 U/L (40–115), but his alanine aminotransferase (ALT) and bilirubin were normal; he denied alcohol use or hepatotoxic medications. Albumin was normal. An erythrocyte sedimentation rate was 67 mm/hr (0–20). Cryptococcal serum antigen, urine histoplasmosis antigen, Bartonella and Brucella serologies, and an interferon-gamma release assay for* M. tuberculosis* were all negative.

**Figure 1:**
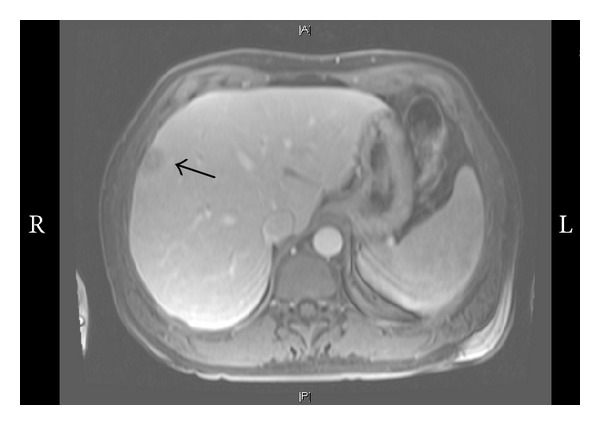
Magnetic resonance of abdomen showing two lesions within the right lobe of the liver along the peripheral surface (black arrows).

**Figure 2:**
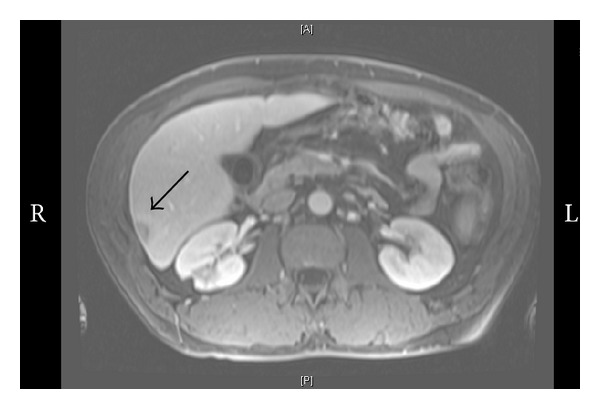
Magnetic resonance of abdomen showing two lesions within the right lobe of the liver along the peripheral surface (black arrows).

**Figure 3:**
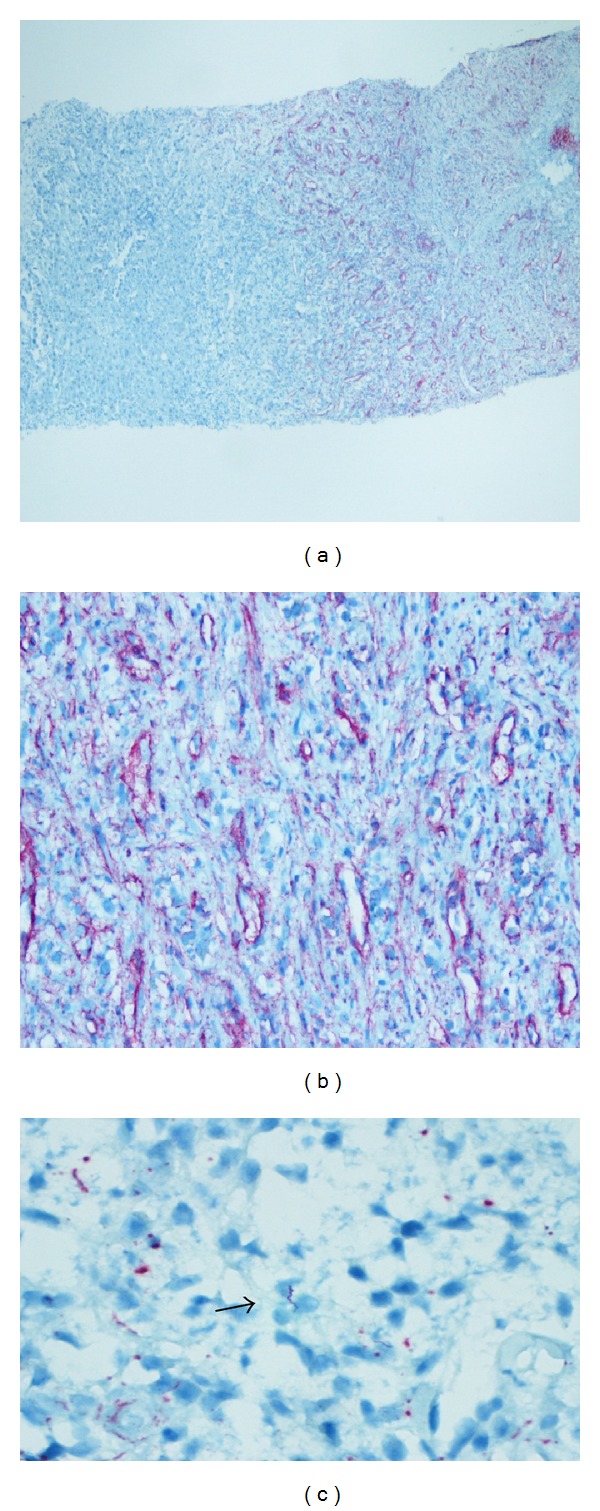
Histological findings: (a) IHC, 40X: scanning power view of liver core biopsy immunohistochemically stained for* Treponema pallidum*. Organisms are visible as red color and correspond to the fibrotic inflamed area seen on hematoxylin and eosin stain (H&E).* T. pallidum*, 40X. (b) IHC, 200X: intermediate power view shows spirochetes centered around blood vessels and infiltrating into sclerotic hepatic parenchyma.* T. pallidum*, 200X. (c) IHC, 600X: high power magnification of spirochete highlights the spiral morphology.* T. pallidum*, (black arrow) 600X.

A computed tomography (CT) of the abdomen pelvis and chest showed a questionable liver abnormality. Magnetic resonance (MRI) of liver confirmed three lesions within the right lobe with mild splenomegaly; lymphoma was considered the most likely diagnosis (Figures 1 and 2). Fine needle liver biopsy demonstrated chronic inflammation and extensive sclerosis; numerous spirochetes were identified on immunohistochemistry (Figure 3). A repeat RPR was 1 : 256; it had been negative four months previously at his annual evaluation.

His visual complaints prompted an ophthalmology evaluation revealing panuveitis in both eyes without retinal involvement, consistent with syphilis. A lumbar puncture showed a lymphocytic pleocytosis with 18 WBC (92% lymphocytes), protein 118, glucose 76, and a reactive VDRL (1 : 1).

Due to his work, he found a continuous penicillin infusion very difficult to be compliant with; therefore, ceftriaxone 2 gram per day for 14 days was chosen for his syphilis therapy. Within hours of the initiation of antibiotic, he developed a Jarisch-Herxheimer reaction with fevers, chills, and tachycardia which resolved within one day. He had complete resolution of systemic symptoms by the end of the course of ceftriaxone with improved vision on the left. One day after discontinuation of ceftriaxone, his right vision worsened acutely. A follow-up evaluation two days after completion of ceftriaxone showed worsening of panuveitis in the right eye with new retinal involvement. A 14-day course of penicillin G (PCN G) 24 million units intravenously every 24 hr was immediately initiated. His right vision improved during his 10 days of high dose penicillin treatment. He was also given weekly benzathine penicillin times 3 doses after the intravenous penicillin, completing a total of 7 weeks of syphilis treatment. HIV treatment was again declined. A repeat CD4 count and HIV-l load at the end of his syphilis treatment were 785 CD4 cell/*μ*L and 792 copies/mL, respectively. His liver function tests normalized after treatment. Nine months after presentation his RPR was 1 : 16 and he was completely well.

## 3. Discussion

Syphilis/HIV coinfection accounts for approximately 25% of the cases of primary and secondary syphilis reported in the United States [5]. In recent years the majority of US syphilis cases have been reported among younger men who have sex with men (MSM); these cases account for 67% of primary and secondary syphilis. These increasing rates of syphilis among young men have been characterized by high rates of HIV coinfection and high risk sexual behavior [6–12].

Syphilis may present with a variety of clinical manifestations, most commonly with a painless chancre at the inoculation site that represents primary syphilis. Approximately 25% of untreated infected individuals will develop a systemic illness or secondary syphilis characterized by a rash, fever, and lymphadenopathy caused by the dissemination and invasion of spirochetes to mucocutaneous and visceral sites. Primary and secondary syphilis can overlap in HIV-coinfected patients as well as in the general population [13, 14].

Though hepatic involvement with syphilis was described over 400 years ago by Paracelsus, Matthiolus, and Francois de la Boe Sylvius [1, 15], syphilitic hepatitis continues to be an under recognized manifestation of syphilis. It is usually defined as the presence of abnormal liver enzymes in the setting of early syphilis and in the absence of an alternative cause of hepatitis that resolves with syphilis treatment. An elevation in the alkaline phosphatase (ALP) during secondary syphilis is the classic presentation in both the general population and HIV-infected patients. While the cholestatic pattern is more common, some cases may present predominantly with hepatocellular damage. The difference in presentation may be dictated by disease stage as suggested by Manavi et al. [4] who found that ALT was higher in proportion to other liver function test values in early syphilis, suggesting that hepatocellular predominant liver injury may be seen with early syphilis and may progress into cholestasis at later stages if left untreated. Elevation of total bilirubin was rarely seen in this cohort and no significant relationship between RPR titer and various HIV factors was observed. Our patient had the classic cholestatic pattern with an abdominal CT as well as MRI revealing three low attenuation lesions in the right lower lobe. Only one case report of pseudohepatic tumor associated to secondary syphilis in an HIV positive patient has been previously described [2].

Liver biopsy in syphilis may be nonspecific, typically characterized by periportal lymphocytic infiltration with focal necrosis of hepatic cells around veins, portal, and lobules. Although the visualization of spirochetes in liver tissue is diagnostic, these are observed in about half of reported cases [1, 16, 17]. Our case showed chronic inflammation and extensive sclerosis with innumerable spirochetes by immunohistochemistry.

Though localized or generalized maculopapular rash is the most common physical finding in patients with syphilitic hepatitis and HIV coinfection, other findings have been described. Crum-Cianflone et al. [3], in a recent retrospective study of twelve HIV/syphilis coinfected patients, reported the most common symptoms included rash (67%), pharyngitis (33%), adenopathy (25%), fever (16%), fatigue (16%), ocular symptoms (16%), and arthralgias (8%).

Mullick et al. [1] reported the first case series of syphilitic hepatitis in seven HIV-infected patients, all men presenting with a rash consistent with secondary syphilis and markedly elevated ALP, which improved significantly after two-week course of intravenous beta-lactam antibiotics. In this study patients with higher CD4^+^ cell counts had higher RPR titers, suggesting that coinfected patient with a preserved immune response may be more likely to develop a robust periportal inflammation responsible for the clinical manifestations, although other studies have showed no correlation between RPR titers and CD4 cell counts or HIV viral load [3, 4].

Although the pathogenesis is unknown, anal intercourse among MSM has been proposed as a risk factor for secondary syphilitic hepatitis, as* T. pallidum* may enter the portal system by direct venous drainage from the rectal area [3, 17]. An anorectal ulcer was not detected in our patient and he denied any intercourse in several years.

The characteristics of HIV-infected patients do not seem to play a role in the occurrence of hepatitis with early syphilis, except for the duration of HIV infection, as reported by Crum-Cianflone et al. [3]. The CD4 cell count, HIV viral load, and use of HAART were not associated with hepatitis. The duration of HIV infection may be of relevance as patients with syphilitic hepatitis typically have a longer duration of infection, 10 versus 4 years (*P* = 0.04). The authors suggested that this association may be the result of an intensified inflammatory response to the treponemal periportal infiltration in the presence of possible subclinical liver disease from cumulative exposure over time. The nature of this relationship is unknown and further studies on the pathogenesis will be needed to confirm this potential association [3, 4].

The incidence and prevalence of syphilitic hepatitis in HIV-infected patients are unknown but it seems more prevalent in men, mostly MSM [3, 4]. The high incidence and prevalence among HIV coinfected MSM may be the result of the higher rates of syphilis among this group. Although anal sex has been proposed as a potential risk factor for syphilitic hepatitis, more research is needed to confirm this hypothesis as syphilitic hepatitis has also been well reported in heterosexual men [1, 17–20].

Most cases show complete clinical and laboratory recovery after syphilis treatment, with normalization of all liver test abnormalities within days or months [3, 4]. These data suggest that syphilitic hepatitis in HIV-infected patients may have the same favorable prognosis as the general population. Liver cirrhosis following hepatic syphilis has been rarely reported, as well as a severe acute and fulminant hepatitis requiring liver transplant, but no such cases have been reported among HIV-coinfected patients. Early recognition and treatment of this condition is needed to avoid potential detrimental health consequences including liver injury, as well as to prevent transmission to others [1, 3, 21–24].

Our case was most unusual in that this patient presented with liver lesions and systemic symptoms highly suggestive of malignancy. A recent RPR had been negative and he denied sexual activity in the preceding 5 years, including oral sex. Syphilis must be entertained as a potential cause of abnormal liver enzymes and/or hepatic lesions, especially in HIV-infected males.
